# Thunderclap-like headache triggered by micturition and angina as an initial manifestation of bladder pheochromocytoma. A case report: Cefaleia trovoada-símile desencadeada pela micção e angina como manifestação inicial de feocromocitoma de bexiga. Um relato de caso

**DOI:** 10.1590/1516-3180.2013.6890002

**Published:** 2014-11-28

**Authors:** You Jin Han, So Young Ock, Eun Jung Kim, Ho Sik Shin, Yeon Soon Jung, Hark Rim

**Affiliations:** I MD. Resident Physician, Department of Internal Medicine, Kosin University College of Medicine, Busan, Korea.; II MD. Assistant Professor, Department of Internal Medicine, Kosin University College of Medicine, Busan, Korea; III MD, PhD. Associate Professor, Department of Internal Medicine, Kosin University College of Medicine, Busan, Korea; IV MD, PhD. Professor, Department of Internal Medicine, Kosin University College of Medicine, Busan, Korea

**Keywords:** Pheochromocytoma, Headache disorders, primary, Micturition, Angina pectoris, Catecholamines

## Abstract

**CONTEXT:**

Pheochromocytoma is a catecholamine-producing tumor characterized by hypertension, headache, tachycardia, excessive diaphoresis and angina. The thunderclap headache is so named because the pain strikes suddenly and severely. Although the symptoms of bladder pheochromocytoma are rather evident, the diagnosis of this rare neuroendocrine tumor can be missed.

**CASE REPORT:**

This study reports the case of a woman diagnosed with bladder pheochromocytoma who experienced thunderclap headache triggered by micturition and angina as an initial manifestation.

**CONCLUSION:**

This case study suggests that thunderclap headache and angina occurring concurrently with sudden blood pressure elevation during or immediately after micturition are important diagnostic clues for bladder pheochromocytoma.

## RESUMO

**CONTEXTO:**

Feocromocitoma é um tumor produtor de catecolaminas, caracterizado por hipertensão, cefaleia, taquicardia, sudorese excessiva e angina. A dor de cabeça trovão é assim chamada porque a dor ataca de repente e intensamente. Embora os sintomas de feocromocitoma de bexiga sejam bastante evidentes, o diagnóstico deste tumor neuroendócrino raro pode ser perdido.

**RELATO DE CASO:**

Este estudo relata o caso de uma mulher diagnosticada com feocromocitoma na bexiga que sentiu a dor de cabeça trovão, desencadeada pela micção e angina como manifestação inicial.

**CONCLUSÃO:**

Este estudo de caso sugere que a dor de cabeça trovão e angina ocorrendo simultaneamente com a elevação da pressão de sangue repentina durante ou imediatamente após a micção são dicas importantes de diagnósticos de feocromocitoma na bexiga.

## INTRODUCTION

Pheochromocytoma, which is a catecholamine-producing tumor that causes secondary hypertension, arises from any location in which the chromaffin cells of the sympathetic nervous system are present.^1^ In adults, approximately 90% of pheochromocytomas arise within the adrenal medulla and about 10% of extra-adrenal pheochromocytomas arise from the chromaffin cells of the paraganglionic system in the bladder, cranium and abdomen.^2^ Bladder pheochromocytoma is an especially rare neuroendocrine tumor that accounts for less than 1% of all pheochromocytomas and less than 0.06% of all bladder tumors.^3^

In most cases, history-taking is the most important tool for preoperatively diagnosing pheochromocytomas that primarily originate from the bladder. An elevated 24-h urinary excretion level of epinephrine and norepinephrine with their metabolites is the confirmatory diagnostic tool.^4^ The typical clinical presentation of bladder pheochromocytoma is macroscopic hematuria, paroxysmal or persistent hypertension, tachycardia, angina, excessive diaphoresis, facial pallor and weakness, and approximately 50% of the patients experience thunderclap headache (TCH) after micturition.^5^^,^^6^

We report the case of a patient diagnosed with bladder pheochromocytoma who had recurrent TCH after micturition and angina.

## CASE REPORT

A 61-year-old woman was admitted at this time with recurrent headaches after micturition and angina. She had experienced syncope during micturition 15 years prior to admission, and she had undergone coronary angiography and ballooning for chest pain, with angina diagnosed three years later (12 years prior to admission). Two years later, in 2002, the patient was found to have a bladder tumor. Imaging was the only form of follow-up performed for the tumor, which remained the same size until 2007. For seven years prior to admission, she experienced recurrent TCH with hypertension immediately after micturition or during micturition, and treatment with antihypertensive medication had not resulted in improvement. There was no known family history of pheochromocytoma.

The findings from physical examination at the present admission, including funduscopic findings, were unremarkable. Her blood pressure was 120/90 mmHg upon admission but became elevated to 230/110 mmHg after micturition. An electrocardiogram revealed a 2-mm elevation on lead V2-3 and T-wave inversion on V2-6 (data not shown). Coronary angiography did not reveal any significant stenosis but a 30% tubular eccentric narrowing feature was found in the proximal left anterior descending artery, which implied minimal coronary artery disease. An echocardiogram showed that the ejection fraction of the left ventricle was 72% with intact wall motion. We suspected bladder pheochromocytoma on the basis of her history and symptoms, such as headache related to micturition and angina.

**Figure 1.**
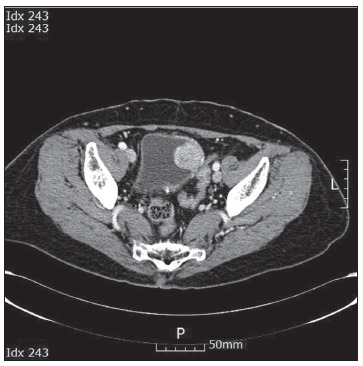
Abdominal computed tomography showing an intraluminal polypoid mass of approximately 3 × 3.2 cm in size with heterogeneous enhancement on the left lateral wall of the urinary bladder.

**Figure 2.**
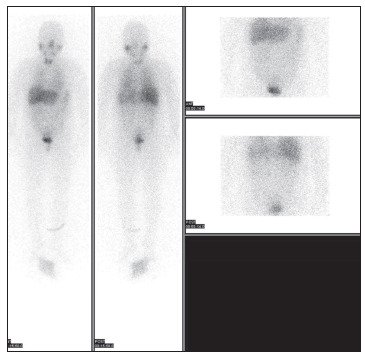
^131^I-MIBG (metaiodobenzylguanidine) scan showing partial uptake on the left side of the urinary bladder, which is consistent with pheochromocytoma.

Her 24-h urinary biochemical analysis showed the following findings: vanillylmandelic acid (VMA), 5.93 mg/day (normal range: 2-8 mg/day); metanephrine, 473 µg/day (normal range: 0-300 µg/day); normetanephrine, 1508 µg/day (normal range: 0-600 µg/day); epinephrine 10.4 µg/day (normal range: 0-20 µg/day); and norepinephrine 171.3 µg/day (normal range: 15-80 µg/day). The blood sampling analysis showed the following results: dopamine, 0.042 ng/ml (normal range: 0-0.2 ng/ml); epinephrine, 0.153 ng/ml (normal range: 0-0.3 ng/ml); norepinephrine, 0.5 ng/ml (normal range: 0-0.8 ng/ml); renin activity, 0.32 ng/ml/h (normal range: 0.1-0.0-6 ng/ml/h); aldosterone, 61.5 pg/ml (normal range: 40-310 pg/ml); adrenocorticotropic hormone (ACTH), 46.3 pg/ml (normal range: 0-80 pg/ml); cortisol, 11.30 ug/dl (normal range: 4.30-22.40 ug/dl); fasting glucose, 103 mg/dl (normal range: 70-124 mg/dl); white blood cell (WBC) count, 6,200; hemoglobin (Hb) 12.2 (normal range: 12.0-16.7 g/dl); and platelets 189,000 (normal range: 144,000-351,000/uL). A contrast-enhanced computed tomography (CT) scan of her abdomen and pelvis revealed a well-enhanced, solid mass of dimensions 3 × 3.2 cm, on the left lateral wall of the bladder (Figure 1). When the CT was performed, the patient’s blood pressure (BP) level was normal. Single-photon emission computed tomography (SPECT) using I-131 metaiodobenzylguanidine (I-131 MIBG) only showed increased uptake in the urinary bladder on its left lateral wall (Figure 2). We diagnosed this case as bladder pheochromocytoma and recommended surgery.

After preoperative treatment with an α-blocker (doxazosin) for two weeks, the patient underwent partial cystectomy under general anesthesia. Immunohistochemical analyses revealed that the tumor cells were positive for CD56, chromogranin and synaptophysin. No remnant tumor was detected on follow-up cystoscopy after surgery, and the patient’s symptoms subsided. She no longer experienced TCH triggered by micturition and angina.

**Figure 3.**
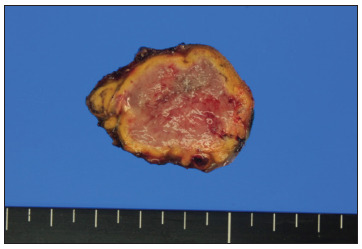
Macroscopically, the pheochromocytoma was a 3 × 3.2-cm sized mass abutting from the left wall of the urinary bladder.

**Figure 4.**
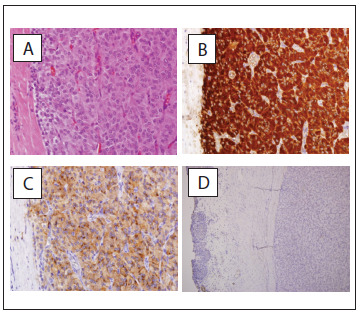
Immunohistochemical analyses revealing that the tumor cells were positive for chromogranin A, S-100 and synaptophysin. (A) Hematoxylin and eosin staining (× 400). (B) Chromogranin staining (× 100). (C) S-100 staining (× 40). (D) Synaptophysin staining (× 400).

Histological examination confirmed the diagnosis of bladder pheochromocytoma with morphological features that indicated benign behavior. Macroscopically, the surgical specimen measured 3 × 3.2 cm in size and the lesion appeared yellow/brown in color and measured 3 cm at the cut surface (Figure 3). Microscopically, the tumor was associated with the muscular bladder wall, whereas the bladder urothelium and the subepithelial stroma were preserved (Figure 4). No evidence of vascular/capsular invasion, necrosis, mitosis or hemorrhage was found. All of the morphological features included in the Pheochromocytoma of the Adrenal gland Scaled Score (PASS) were evaluated, resulting in a PASS score of < 2/20 (tumor with benign behavior).^3^ Immunohistochemical analysis demonstrated positivity for chromogranin A, S-100 and synaptophysin.

## DISCUSSION

Paragangliomas are rare neuroendocrine neoplasms that develop from the germinal cells of the neural crest and usually involve the adrenal glands. Bladder pheochromocytoma is a particularly unusual form of paraganglioma that represents less than 1% of all pheochromocytomas.^7^ A systematic survey of indexed articles using the terms “pheochromocytoma” and “thunderclap headache” in Medline and Cochrane Library databases and MeSH (Medical Subject Headings) revealed that only five articles have been published on this topic to this date. All of these papers were found in Medline, Embase, Lilacs and the Cochrane Library (Table 1).^1^^,^^2^^,^^3^^,^^4^^,^^5^^,^^6^^,^^7^^,^^8^^,^^9^^,^^10^^,^^11^^,^^12^^,^^13^^,^^14^^,^^15^^,^^16^^,^^17^^,^^18^^,^^19^^,^^20^^,^^21^

**Table 1.**
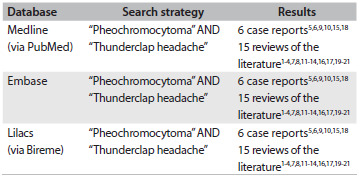
Results from our reviews of medical databases using descriptors for the main clinical findings observed in our patient: February 1, 2013

The clinical features of bladder pheochromocytoma are various and include hematuria and hypertensive crisis together with headache, angina, palpitation, hot flashes and sweating. These crises are typically induced by micturition or overdistension of the bladder, which leads to systemic release of catecholamines. However, not all bladder pheochromocytomas result in this syndrome and some show no hormonal activity. Signs of hormonal activity are seen in 83% of bladder pheochromocytomas in the literature.^8^ In these cases, the diagnosis can be completely incidental. Therefore, detecting the disease requires a high degree of clinical suspicion based on the patient’s symptoms, owing to both the rarity of this lesion and the variety of its clinical features.^9^

TCH is a hyper-acute, severe headache that reaches maximum intensity at onset. The term TCH was first used to describe the headache caused by unruptured intracerebral aneurysms, and patients frequently describe this type of headache as the worst headache of their lives.^10^

This sudden, severe headache peaks within 60 s and usually fades over several hours.^11^ TCH may be an indication of a medical emergency, such as aneurysmal subarachnoid hemorrhage, pituitary apoplexy, carotid or vertebral artery dissection, acute hypertensive crisis and cerebral venous sinus thrombosis, which often leads to death or severe disability.^12^ Therefore, immediate medical attention is needed for any headache that comes on suddenly and severely. In our case, the patient’s usual systolic BP and diastolic BP were 100-110 mmHg/60-70 mmHg, but the systolic BP increased to an average of 180-200 mmHg and the diastolic BP to 100-120 mmHg after micturition. The underlying cause of recurrent TCH with these hypertensive crises was bladder pheochromocytoma, which can lead to a potentially lethal condition when unrecognized.

The diagnosis of pheochromocytoma is supported by measurements of catecholamines and their metabolites metanephrine and normetanephrine, in plasma and 24-h urine samples.^7^ In a study conducted on patients with extra-adrenal pheochromocytomas, the urinary metanephrine rate was high in 88% of the cases.^13^ In our case, the level of 24-h urine metanephrine became elevated to 473 µg/day. After biochemical tests, imaging techniques such as CT or magnetic resonance imaging should be performed to locate the neoplasm. The diagnosis can be enhanced by iodine-MIBG scanning, which has a sensitivity of 78% for adrenal pheochromocytomas and 67-89% for extra-adrenal locations.^8^

We used contrast-enhanced CT scans and iodine-MIBG scanning to diagnose and determine the location of the tumor. To date, no definitive biochemical or histological markers have been defined for distinguishing benign from malignant tumors. The only definitive proof of malignancy is the presence of metastasis to other organs.^14^ No standardized histological features or any scoring system to distinguish benign from malignant bladder pheochromocytomas have yet been reported in the literature.

The PASS system, which is usually used to study adrenal gland pheochromocytomas,^3^ was applied to determine whether the tumor had benign or aggressive behavior in the present case. Application of these criteria to a larger series of bladder pheochromocytomas may help to evaluate the presence of malignant bladder pheochromocytoma. The PASS system can be applied to bladder pheochromocytomas as well as to adrenal gland pheochromocytomas.

This case showed a PASS score of < 2/20, thus indicating benign tumor behavior. Microscopically, bladder pheochromocytomas produce submucosal or intramucosal masses; furthermore, they are covered by intact epithelium and are characterized by the presence of nests (“Zellballen”) or cords of cells delimited by fibrovascular connective tissue. The majority of the cells are medium-to-large and polyhedral, and have eosinophilic cytoplasm and ovoid nuclei. The unclear chromatin is dispersed and one or more nucleoli are generally present. Large bizarre nuclei may be seen.^15^ In our case, these cells involved the true muscle, submucosa and mucosal layer with positivity for chromogranin A, S-100 and synaptophysin, and were confirmed as bladder pheochromocytoma.

Bladder pheochromocytoma is an extremely rare disease.^16^ Because of its rarity, diagnosing bladder pheochromocytoma preoperatively may be difficult and delayed, in spite of the characteristic diagnostic clues such as paroxysmal hypertension and headache immediately after micturition.^17^ Diagnostic delays as long as several years from the onset of symptoms have been reported, which can probably be attributed to the rarity of the tumor.^18^ Although laboratory and radiological investigations are informative and necessary for diagnosing bladder pheochromocytoma, a high degree of suspicion based on a detailed history and physical findings is very important. Such suspicions facilitate early diagnosis and treatment of the tumor, which leads to a good outcome.^18^

In our case, a detailed history that included headache-provoking factors and detection of the transient hypertension and angina induced by micturition provided early diagnostic clues for this rare disease. Through recognizing the relationship between headache and micturition, we were alerted to the possibility of bladder pheochromocytoma in this case. Common presenting features of bladder pheochromocytoma include symptoms of painless hematuria, diaphoresis, hypertension, angina, headache, palpitation and fainting after micturition, caused by catecholamine release.^16^

In this case, blood pressure elevation was apparent following micturition and activities with the capability of increasing the pressure inside the abdomen, which would thus push down on the bladder. Imaging studies are useful for planning definitive treatment because they can determine both the tumor location and whether multiple sites of involvement or metastases exist.^17^

In our case, the diagnosis was established by finding increased levels of catecholamine metabolites in the urine and CT imaging after clinical suspicion. The optimal treatment for pheochromocytoma is prompt surgical excision, because these patients are at significant risk of lethal complications such as hypertensive crisis.^17^ Patient preparation is an essential step and includes preoperative treatment with α- and β-blocking agents.^19^ Open surgery to perform partial cystectomy is recommended because of the multilayer involvement of the bladder wall. Radical cystectomy with pelvic lymphadenectomy is recommended if metastasis is definitely present.^8^ Radiotherapy and chemotherapy have limited effectiveness.

Extra-adrenal pheochromocytomas are more likely to recur and metastasize than their adrenal counterparts, thus making long-term follow-up with annual determinations of catecholamine production essential because it is not histologically feasible to differentiate benign from malignant pheochromocytomas.^17^

Long-term follow-up with regular biological and clinical examinations is also necessary in order to detect recurrence or metastasis. Although no standard criteria for malignancy exist from a histological perspective, appearance of a metastasis is a sign of malignancy.^20^

Of the 170 cases of bladder pheochromocytoma that have been reported so far, only 17 well-documented cases were malignant.^21^ Thus, the prognosis for bladder pheochromocytoma remains favorable.

## CONCLUSION

In summary, this case serves as a reminder of the importance of thorough history-taking and clinical examination in making diagnoses. Nephrologists, endocrinologists, cardiologists and urologists should work together to minimize the risk of misdiagnosis. This case study suggests that TCH and angina occurring concurrently with sudden blood pressure elevation during or immediately after micturition are important diagnostic clues for bladder pheochromocytoma.
